# Quantifying the recovery process of skeletal muscle on hematoxylin and eosin stained images via learning from label proportion

**DOI:** 10.1038/s41598-024-78433-z

**Published:** 2024-11-07

**Authors:** Yu Yamaoka, Weng Ian Chan, Shigeto Seno, Kanako Iwamori, So-ichiro Fukada, Hideo Matsuda

**Affiliations:** 1 Graduate School of Information Science and Technology, Osaka University, Osaka, 565-0871 Japan; 2 Graduate School of Pharmaceutical Sciences, Osaka University, Osaka, 565-0871 Japan

**Keywords:** Skeletal muscle, HE Stain, Weakly supervised learning, Whole slide image (WSI), Learning Label Proportion (LLP), Scientific data, Computer science, Image processing, Muscle stem cells

## Abstract

Visual observing muscle tissue regeneration is used to measure experimental effect size in biological research to discover the mechanism of muscle strength decline due to illness or aging. Quantitative computer imaging analysis for support evaluating the recovery phase has not been established because of the localized nature of recovery and the difficulty in selecting image features for cells in regeneration. We constructed MyoRegenTrack for segmenting cells and classifying their regeneration phase in hematoxylin–eosin (HE) stained images. A straightforward approach to classification is supervised learning. However, obtaining detailed annotations for each fiber in a whole slide image is impractical in terms of cost and accuracy. Thus, we propose to learn individual recovery phase classification utilizing the proportions of cell class depending on the days after muscle injection to induce regeneration. We extract implicit multidimensional features from the HE-stained tissue images and train a classifier using weakly supervised learning, guided by their class proportion for elapsed time on recovery. We confirmed the effectiveness of MyoRegenTrack by comparing its results with expert annotations. A comparative study of the recovery relation between two different muscle injections shows that the analysis result using MyoRegenTrack is consistent with findings from previous studies.

## Introduction

Skeletal muscles possess repair capabilities^1^ and can restore muscle function that has declined due to injury or disease. Previous studies investigated this regeneration process with tissue regeneration models^2,3^. In skeletal muscle research, evaluating tissue regeneration is necessary to measure the effect size of experimental perturbation^4^. Since the morphology of tissues reflects the influence of injuries and diseases, it has been used as an indicator to monitor the health status of skeletal muscles^5^. One method used to observe the morphology of myofibers during regeneration is cardiotoxin (CTX) injection, where CTX is injected into the lower leg muscles of mice to induce necrosis of myofibers for subsequent regeneration of muscle tissue over several days to weeks. CTX is a type of snake venom from cobras (Naja pallida, Naja nigricollis) that selectively damages only myofibers through its toxins^6^. In this process, muscle satellite cells and the basal membrane, which includes blood vessels, nerves, and collagen, remain intact, thus facilitating swift regeneration. Myofibers, the primary cells constituting muscle tissue, undergo necrosis when damage occurs. Subsequently, inflammatory cells infiltrate the tissue and remove the necrotic myofibers, leaving behind only the basal membrane known as ghost fibers^7^. Damaged-myofiber-derived factors (DMDFs) from the necrotic myofibers^3^ induce the activation of muscle satellite cells, which proliferate and become myoblasts. Proliferated myoblasts cease cell division during regeneration and fuse with adjacent myoblasts to form multinucleated myotubes. As the myotubes further mature, they grow into myofibers. When regeneration is complete, the mature myofibers fill the gaps and crowd together within the tissue. However, regeneration does not progress uniformly throughout the entire muscle tissue^8,9^. The phase of regeneration varies by region, which may lead to incorrect interpretations depending on the analyzed location. It is necessary to comprehensively analyze the entire muscle tissue rather than focusing on localized myofibers, but wide-field observation is labor-intensive. Therefore, research has been advancing towards automating objective and quantitative computer-based image analysis^10–22^.

Most previous studies focused on images of intact muscle tissues stained with laminin instead of hematoxylin–eosin(HE) stained images during recovery. Although laminin staining is suitable for visualizing cell membranes and facilitates the segmentation of stable tissues, it is close to binarization of color information in the captured images, which leads to a lack of image features related to cell texture for verifying the regeneration phase and, thus, causes a risk of incorrect classification of ghost fibers, myoblasts, myotubes, and other cells as intact myofibers when analyzing tissues during recovery. On the other hand, compared with cells stained with laminin, cells stained with the most common and convenient HE stain can capture various color, peripheral, and contour information compared to laminin staining, particularly the maturation and distribution of myofibers and the hazy contours of ghost fibers, making it suitable for evaluating the regeneration phase of each fiber. In other words, the increased color and peripheral information, along with the less distinct cell membrane edges, raise the complexity of computational analysis. Previous studies have dealt with the segmentation of laminin-stained^10–19^, HE-stained^21,22^, and Picrosirius red stained^20^ intact myofibers, but excluded HE-stained myofibers from early damage to late recovery from image analysis due to their complexity. Addressing this issue involves two steps: the first is the segmentation of cells other than myofibers, and the second is the classification of the recovery phase. We developed methods for segmenting other cells in recovery, such as myoblasts and ghost fibers, while previous studies^22^ have successfully developed only for intact myofibers in HE-stain. Moreover, the explicit features obtained from segmentation are insufficient for classifying various cells during recovery^17,23^. Some image analysis used to classify myofiber includes Myosoft^17^, MuscleJ^11^, and Open-CSAM^15^, which target laminin stained muscle tissues instead of HE-stained ones and extract features such as area and circularity from segmented myofibers. Open-CSAM^15^ calculates the cross-sectional area (CSA) for laminin-stained images from day 8 to day 28 after a CTX injection. Users need to manually set the cell size and circularity thresholds according to the elapsed days, mouse age, and health condition, and misdirected or undetected fibers must be added manually. This process is influenced by the user’s level of expertise and the imaging conditions. Myosoft^17^ also uses shape-related features such as the Feret aspect ratio and minimum Feret distance to determine fiber types. This approach lacks robustness against domain gaps because manual feature selection relies on domain knowledge. We conducted the recovery phase of cell classification using a support vector machine and confirmed that the manual features selected in Myosoft were not applicable for HE-stained images during recovery, as shown in Supplementary Fig. S1. Manual explicit feature selection is not applicable when no explicit features indicate the phase, such as the embryonic/neonatal myosin heavy chain from day 3 to day 8 during recovery^4^. In contrast, implicit features in machine learning offer strong expressiveness with more dimensions than manually selected features, enabling adaptation to a wide range of domains.

In this study, we use a pretrained DINO^24^ vision transformer model as the feature extractor for unlabeled cell images, which is a vision foundation model using self-supervised learning and provides greater accuracy than CellProfiler^25^ in the tasks of drug target and gene family classification. We then use a multilayer perceptron^26^ consisting of fully connected layers and rectified linear unit (ReLU)^27^ activation functions to classify recovery phase of cells using the obtained features. In learning a multilayer perceptron, we do not use supervised methods^28,29^, which use ground truth labels during training, because the annotation cost is high and it is not easy to ensure the accuracy of the annotations. Instead, we consider leveraging the time series data (days elapsed since CTX injection), the only available prior information, as weak supervision to perform classification tasks. Given the limited GPU memory, a whole slide image (WSI) of muscle tissue is clipped per segmented cell. We classify the segmented cells into four classes of recovery phase: stable (intact or complete regeneration), early phase, mid-phase, and late phase. Assigning classes based on a clipped image is challenging, and it is easier to derive the proportion of all classes in a WSI via the general observation of muscle tissue. For example, on day 0, all fibers are stable as CTX was not injected prior; on day 3, ghost fibers and myoblasts are abundant. Over time, the number of myotubes increases while the number of myoblasts decreases. The most common method for training a model from class proportion data is pseudo-labeling^30^. Given the class proportions of cells associated with specific daily labels, we can assign a probable class to a clipped image based on these proportions. Once these classes are assigned, we can train a classifier using the traditional supervised learning approach, where the classifier learns from the pseudo-labeled images. However, pseudo-labeling^30^ can introduce errors during training, which may prevent the classifier from achieving high accuracy. Therefore, we propose to use learning from label proportions (LLP)^31^, a weakly supervised learning method that utilizes the class proportions of groups of multiple instances, even if individual instances are unlabeled. During our training with LLP, the classifier model predicts the classes of a group of multiple cell images clipped from a WSI associated with a label of day elapsed since CTX injection, where we can compute the predicted class proportion of the group, and optimize a loss function by comparing these predicted class proportions with the true class proportion associated with predefined daily labels. LLP has demonstrated strong performance in medical database labeling, for which generating individual instance labels is difficult due to privacy concerns, such as estimating individual embryo implantation success rates using the actual implantation ratio data of patient group in embryo selection for improving pregnancy rates^32^. LLP is also used for medical images, particularly when dealing with WSIs with tens to hundreds of millions of pixels. Annotating WSIs at the pixel level for various cell classes is burdensome for specialists and, thus, rarely performed. In the context of WSIs, Ye *et al*.^33^ proposed applying LLP to multiple learning instances for cases in which individual regions or clipped images lacked label annotations for cancer tumor detection. By applying LLP with fuzzy proportions for WSIs, they observed an improvement in the concordance index of the slide necrosis score. There were significant differences in patient prognosis among the groups classified based on the estimated necrosis rate.

In our study, we developed MyoRegenTrack software to inspect the muscle tissue recovery process from HE-stained images visually. This software integrates various machine learning and image processing techniques, including LLP. The software successfully evaluates the overall regeneration phase of muscle tissue by using WSIs of HE-stained muscle tissue as input. Additionally, in experiments evaluating muscle tissue regeneration using CTX and glycerol, the conclusions derived by MyoRegenTrack are consistent with the findings of previous studies^19,34–38^, showing the reliability of MyoRegenTrack.

## Related work

This section introduces related work on computer analyses of muscle tissue. As indicated in Table 1, computational image analyses of muscle tissue have mainly focused on laminin-stained images^10–19^. This is primarily because analysis of muscle tissue often requires calculating the cross-sectional area (CSA). Laminin staining delineates the edges of healthy myofibers, making segmentation easier ith computer algorithms^10,11,13,15–18^ or software such as cellprofiler^12,14,39^ and cellpose^19,40^ in the binarized images. The segmentation data are used to classify between fiber types and identify whether the mouse is a Duchene muscular dystrophy mouse (mdx) based on explicit features of cells such as circularity, area, and feret ratio obtained. SMASH^10^, MuscleJ^11^ and Muscle2View^14^ classify myofibers into various types like slow-type oxidative myofibers and fast-type glycolytic myofibers, using explicit features obtained through segmentation. OpenCSAM^15^ focuses on the accuracy of laminin-stained myofiber segmentation, particularly on tissues subjected to necrosis and recovery induced by CTX. MyoView^18^ is used to observe tissues from mice that undergo high-intensity interval training, showing that the average CSA of myofibers over time can be segmented with an accuracy equivalent to that of manual methods. MyoView^18^ has also achieved the highest accuracy in segmenting intact muscle fibers, compared to other segmentation tools such as openCSAM^15^, MuscleJ^11^, SMASH^10^, and MyoVision^13^. Myosoft^17^ has been used to successfully classify myofibers based on metabolic and contractile properties using features such as area, circularity, and minimum Feret diameter. As shown in Supplementary Fig. S2, since it is difficult to apply software developed for laminin staining to other staining methods, alternative software such as LabelsToRois^19^, Laghi *et al*.^20^, Liu *et al*.^21^, and MyoSOTHES^22^ has been investigated. Both LabelsToRois^19^ and MyoSOTHES^22^ are based on cellpose^40^, which is also treated as a foundational model in our approach. LabelsToRois has validated the segmentation capability of cellpose using laminin, phalloidin, and WGA staining, confirming that it achieves accuracy comparable to expert manual segmentation. Notably, MyoSOTHES^22^ focuses on HE-stained images, the same as ours, and performs segmentation that underpins the evaluation of recovery progress targeted in our study. However, their focus has been limited to myofibers while we segment other cells, such as ghost fibers, myoblast, and myotubes that appear during recovery. Explicit features obtained from segmentation alone that have been used in previous studies were insufficient for analysis in regeneration processes. Therefore, we propose a new classification method of recovery phases using implicit features of machine learning techniques DINO^24^.

**Table 1:** Related work of computing myofiber. It is important to note that segmentation is performed before classification. “mdx” refers to mice with Duchene muscular dystrophy. The bold text in the table indicates a new suggestion.

Name	Stain	Target	Analysis	Method
SMASH^10^	Laminin	Intact, mdx	Classification (fiber types)	Algorithm
MuscleJ^11^	Laminin	Intact, mdx	Classification (fiber types)	Algorithm
MuscleAnalyzer^12^	Laminin	Intact, mdx	Segmentation	CellProfiler^39^
MyoVision^13^	Laminin	Training, disuse atrophy	Segmentation	Algorithm
Muscle2View^14^	Laminin	Intact	Classification (fiber types)	CellProfiler^39^
OpenCSAM^15^	Laminin	Damaged (days 0,8-24), mdx	Segmentation	Algorithm
MyoSight^16^	Laminin	Intact, mdx	Segmentation	Algorithm
Myosoft^17^	Laminin	Intact, mdx	Classification (fiber types)	Algorithm
MyoView^18^	Laminin	Training (days 0,28,56)	Segmentation	Algorithm
LabelsToRois^19^	Laminin, etc.	Intact, mdx	Segmentation	Cellpose^40^
Laghi *et al*.^20^	Picrosirius red	Intact, mdx	Segmentation	CellProfiler^39^
Liu *et al*.^21^	HE	Intact, mdx	Segmentation	Algorithm
MyoSOTHES^22^	HE	Intact, mdx	Segmentation	Cellpose^40^
**Ours**	HE	Damaged (days 0,**3-14**)	Classification **(recovery phases)**	Cellpose^40^, **DINO**^24^

## Results

**Fig. 1:**
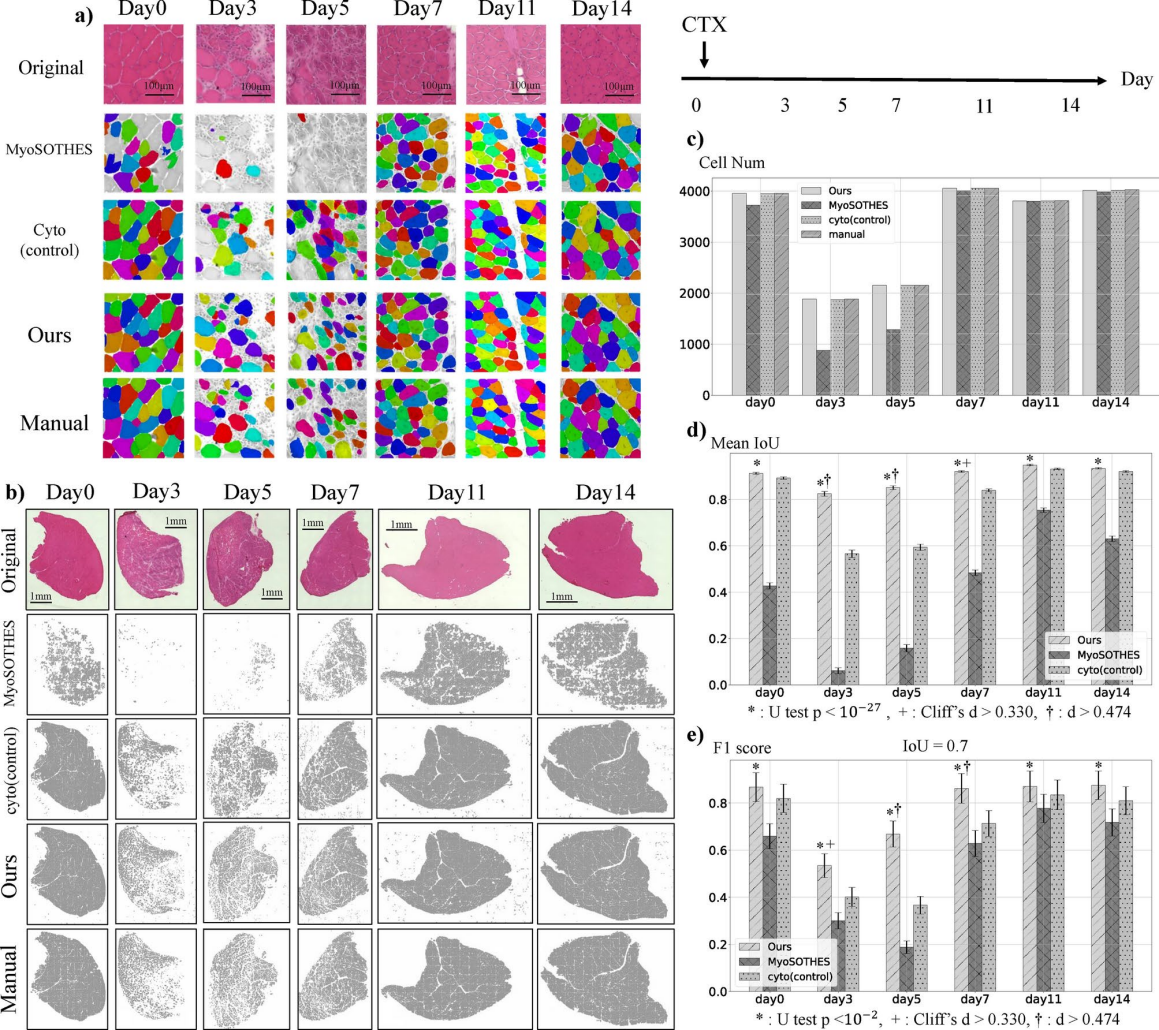
Segmentation results. The results from three models-MyoSOTHES^22^, cyto (a pretrained model provided by Cellpose^40^), and Ours (our finetuned model)-are compared against Manual (manually segmented ground truth data). The results are displayed according to the number of days elapsed since the CTX injection. Note that day 0 refers to the period before the CTX injection.  (**a**) Example of the results after processing the $256 \times 256$ [pixel] square images with the model. Different colors indicate different myofibers.  (**b**) The results of WSI processing. When input into the model, an image was divided into $256 \times 256$ [pixel] square images. The obtained segmentation data were then reassembled in their original order to produce the combined result.  (**c**) Cell Num: Indicates the number of cells (objects) segmented by each model.  (**d**) If there were no overlapping cells, indicating that the cells were undetected, the IoU was considered 0. The error bars represent the 95% confidence interval. *: Mann-Whitney U test (two sided test) $p<10^{-27}$, †: Cliff’s $d>0.474$^41^. The sample size is the CellNum of each day.  (**e**) The F-score is computed for each $256 \times 256$ [pixel] square images. Segmentations where the IoU with the Manual was 0.7 or above are counted as true positives (TP). If no myofibers are in the Manual with an IoU of 0.7 or above within the predicted area, it is considered over-detection and marked as false positive (FP). If a myofiber present in the Manual result was not segmented, it is counted as a false negative (FN). The error bars represent the 95% confidence intervals. *: Mann-Whitney U test (two sided test) $p<10^{-11}$, +: Cliff’s $d>0.330$, †: Cliff’s $d>0.474$. The Sample size is 100-124.

**Fig. 2:**
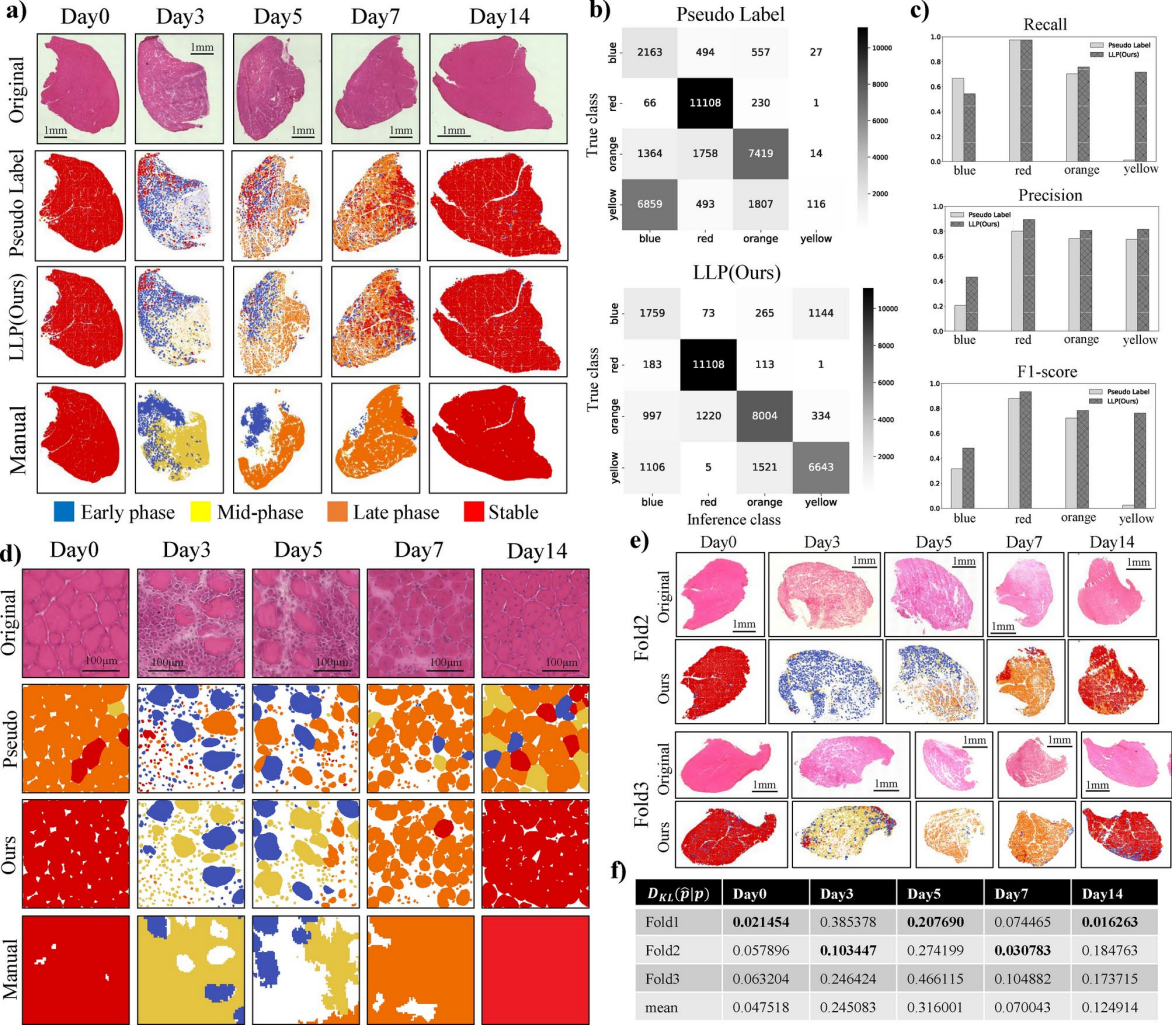
(**a**) Classification results for whole slide images (WSIs). The integrated results from the $256 \times 256$ [pixel] square images shown in (**d**) are presented. Each color was defined as follows: red: stable, blue: early phase, yellow: mid-phase, orange: late phase. The “Original” represents the initially captured raw images. “Pseudo Label” shows the results using class proportion information to assign pseudo labels^30^. “LLP” refers to training using the Proportion Loss as shown in Fig. 6(c). “Manual” indicates expert manual class annotations, with white areas representing indeterminate regions. (**b**) The true class (Manual) is shown in the horizontal direction, and the inference results are shown in the vertical direction. (**c**) From the results of the confusion matrix, performance metrics such as precision (how accurate the inferences are), recall (how well the model captures all true instances), and F1-score (the harmonic mean of precision and recall) are calculated. Values close to 1 indicate good model accuracy. **d**) Inference results for each day based on $256 \times 256$ [pixel] square images. Cellpose^40^ was used to conduct segmentation tasks with settings of Diameter=5 or None (default), and each cell was cropped into a 64px square image for feature extraction and class inference as shown in Fig. 6(a). The results colored with the Diameter=5 setting are labeled as Layer 0, and those colored with the Diameter=None setting are labeled as Layer 1. The results of the $256 \times 256$ [pixel] square images are obtained by overlaying these two layers, prioritizing the results of Layer 1. (**e**) Presentation of the 3-fold cross-validation results. Fold1 corresponds to the results in (**a**) since there are no detailed Manual annotations by experts for Fold2 and Fold3. The color class correspondence and integration method are the same as in (**a**, **d**). (**f**) Quantitative evaluation of the cross-validation. The class proportions are calculated for each Test image in each fold, and the KL divergence between these proportions and those calculated from the inference results in (**e**) are shown for each fold and on average. A smaller KL value indicates a closer approximation to the true distribution.

**Fig. 3:**
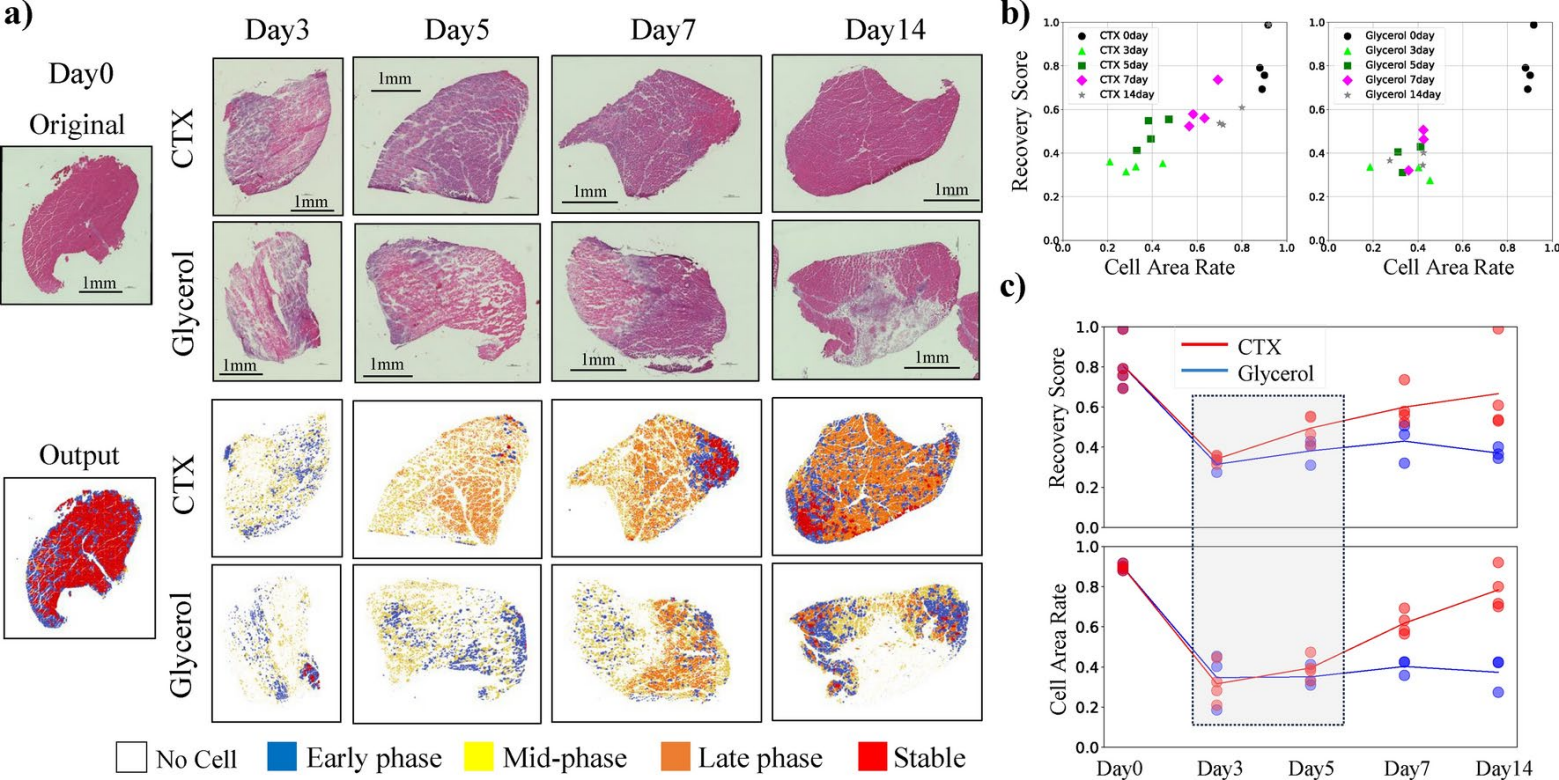
The results of inputting images of tissues injected with cardiotoxin (CTX) or glycerol into the model trained using LLP, as shown in Fig. 2. (**a**) Day 0 represents the tissue before injection. Days 3 and beyond indicate the number of days elapsed since the injection of CTX or glycerol, which induces necrosis and recovery in the tissue. The output displays the results from the proposed software, where each color corresponds to red: stable, blue: early phase, yellow: mid-phase, orange: late phase and white indicates areas where no cells were detected. (**b, c**) We count the pixels of each color within the edge-detected tissue and compute the proportion $\hat{p}$ of each color relative to the Cell Area (Segmentation Area). Each image’s recovery score was calculated after determining $\omega$ using the procedure in Equation 4. Each point in this figure corresponds to one WSI. The number of images for each date can be found in Table 2 under unlabeled test data (CTX) and (glycerol). The cell area is calculated by dividing the cell segmentation area by stain area, indicating the area ratio of cells detected by Cellpose^40^ to the muscle tissue region. Note that since Day 0 was before injection, the data for CTX and glycerol are aligned.

To demonstrate the capability of MyoRegenTrack in evaluating muscle tissue regeneration, we conducted three experiments: segmentation of HE-stained images, comparison with expert manual class annotation classification, and analysis of CTX or glycerol injection different recovery processes. In the fine-tuning of Cellpose^40^, pretrain model of cell segmentation used with MyoSOTHES^22^, we prepared images from days 0, 3, 5, 7, 11, and 14 of mouse muscle tissue recovery following cardiotoxin (CTX) injection. We refined the model through fine-tuning and accurately segmented ghost fibers, myoblasts, and myotubes observed on days 3 and 5, confirming that our method provides higher accuracy than methods used in prior studies^22^ and the baseline^40^, as shown in Fig. 1. In muscle classification, we perform class inference using a classifier model developed through feature extraction and the learning from label proportion (LLP) method. We verify the accuracy of the proposed method by coloring each class and comparing it with expert manual class annotations, as well as through cross-validation as demonstrated in Fig. 2. In the comparison of recovery following the injection of glycerol or CTX, we assessed the ability of the proposed software to automatically detect the well-known inhibitory recovery effects of glycerol, thus verifying the usefulness of detailed analysis through classification, as shown in Fig. 3.

### Fine-tuning of Cellpose with segmentation

Fig. 1 presents the results of the segmentation analysis of muscle tissue images from days 0, 3, 5, 7, 11, and 14. Day 0 represents the time before the injection of CTX, and the subsequent days indicate the number of days after the CTX injection. Fig. 1(a,b) are visual qualitative evaluations of segmentation, with (a) showing cell-level details and (b) depicting the entire muscle tissue in a whole slide image (WSI). Fig. 1(c-e) represents the quantitative evaluation of segmentation. All images input into Cellpose were evenly clipped from a whole slide image (WSI) into $256 \times 256$ [pixel] square images in the grid-like arrangement, with examples of the results displayed in Fig. 1(a). The reassembled results of each 256 square image onto the WSI are shown in Fig. 1(b). The number of objects segmented during inference and manually segmented objects are shown in Fig. 1(c). Fewer detections than manual results reflect underdetection, while more detections reflect over-detection. To assess the model’s performance with manual segmentation results as the ground truth data, we used the average overlap of the predicted and true values, the mean IoU (Fig. 1(d)), and F1-score in Fig. 1(e). Mean IoU represents the average intersection over union (IoU) of the predicted segmentation of each cell compared to the manually established ground truth segmentation, averaged over the number of cells (CellNum). The calculation of the F1-score, the harmonic mean of precision and recall derived from this counting method using the confusion matrix, was based on the approach used in MyoSOTHES^22^, where precision and recall were computed for various IoU thresholds before being combined, with the value at an IoU threshold=0.7 shown. Each test was controlled based on the results of the cyto model^40^.

### Classification of muscle recovery status

Using whole slide images (WSI) of the recovery process from days 0, 3, 5, 7, and 14 following the injection of mouse muscle tissue with CTX, we validated the accuracy of the proposed method by comparing the results to expert manual class annotations as shown in Fig. 2(a-d). Fig. 2(a,d) are visual qualitative evaluations of segmentation, with (d) showing cell-level details and (a) depicting the entire muscle tissue in a whole slide image (WSI). All images were uniformly clipped from the WSIs into $256 \times 256$ [pixel] square images for input into the DINO feature extractor and classifier, with examples of these results displayed in Fig. 2(d). The results of the reassembly of each $256 \times 256$ [pixel] square image for a WSI are shown in Fig. 2(a). Fig. 2(b,c) represents the quantitative classification evaluation. The class inference was performed for each object detected by Cellpose, and the results were compared to manually assigned classes, as summarized in a confusion matrix shown in Fig. 2(b). Recall, precision, and F1-score for each class were calculated from the data in this table as shown in Fig. 2(c). Furthermore, 3-fold cross-validation and swapping of the training and test data were performed to verify the generalization performance, as shown in Fig. 2(e,f).

### Comparison of the recoveries of glycerol and CTX

It was previously reported^19,34–38^ that there are significant differences in tissue necrosis and regeneration between injections of glycerol and cardiotoxin(CTX), with the known appearance of adipocytes due to the inhibition of regeneration after glycerol injections refer to Supplementary Fig. S3. Therefore, we used our MyoRegenTrack software, which is based on LLP, to analyze mice’s muscle tissue images over recovery after the injection of either glycerol or CTX, as shown in Fig. 3. We also provide the analysis results in Supplementary Fig. S4 by Lee *et al*.’s model training method based on pseudo labels^30^. The original WSIs were input into MyoRegenTrack, and the output results for CTX and glycerol are displayed separately in Fig. 3(a). Attention to one sample from day 0 with extensive freezing artifacts was excluded from the analysis (see Supplementary Fig. S5). The recovery score equation(4) was introduced, and, together with the cell area rate obtained from segmentation, it was displayed via a two-dimensional map as shown in Fig. 3(b). Additionally, Fig. 3(c) displays the recovery progression in recovery score and cell area rate separately over time, demonstrating that MyoRegenTrack can delineate the differences in recovery progression between CTX and glycerol. By day 5, although no differences were observed in the cell area rate alone, the recovery score revealed significant differences between the two groups, thus providing detailed analytical information and the area ratios calculated solely from segmentation results.

## Discussion

We discuss the results of cell segmentation and the classification of the recovery phase. We also compared the muscle recovery process after injecting CTX and glycerol, respectively, to evaluate the effectiveness of our method.

The results in Fig. 1 are used to evaluate the performance of the finetuned cell segmentation model adapted to the regeneration process of myofibers. In Fig. 1(a, b) for Days 0, 11, and 14, stable myofibers have been appropriately segmented by both prior studies^22,40^ and ours. However, ghost fibers, myoblasts, and myotubes seen on days 3, 5, and 7 were not recognized as cells by MyoSOTHES^22^ and cyto (Cellpose^40^ pretrain model). Still, ours has significantly succeeded in segmentation compared to previous models, as shown in Fig. 1(a, b)-Day 3,5,7 and (d, e)-Day 3,5,7 and effect size in Mean IoU by chiff’s delta based on the cyto model (control) as follows: $d = 0.574> 0.474$ in day3, $d=0.703>0.474$ in day5, and $d=0.464>0.330$ in day7. In Chiff’s delta, 0.330 corresponds to Cohen’s d of 0.5, indicating a medium effect size and 0.474 corresponds to Cohen’s d of 0.8, indicating a large effect size^41^. This confirms that the segmentation model can detect ghost fibers, myoblasts, and myotubes accurately close to manual detection. by fine-tuning Cellpose. However, it is observed in Fig. 1(b)-Ours-Day7,11,14 that cells are also detected in non-stained parts of the image in our method, indicating an overdetection tendency, especially when detecting myoblasts. This could be mitigated by incorporating an edge detection script for the stained areas, which would disregard detections in blank areas, although this leaves some questions regarding the segmentation model’s performance and reliability limitations. Providing training data that explicitly includes areas without cells might resolve this issue.

Analyzing the tissue recovery process using WSIs, as shown in Fig. 2(c), is suitable for obtaining a macroscopic overview of the tissue but not for discriminating the phases of individual myofibers. Conversely, the use of grid images, as shown in Fig. 2(b), makes it easier to view the phase of each myofiber, but a complete view of the entire tissue is not provided. One solution to this issue is to display all clipped images, although not shown due to space constraints. Notably, there has been a demand for methods that can be used to quantitatively analyze the overall phase of myofiber recovery across the entire tissue. Classification offers one solution, allowing for a comprehensive approximation of the general trends in small areas. Our proposed method LLP yields judgments closer to those of experts than those of the conventional pseudo-labeling^30^ method. However, the current approach combining segmentation and classification does not color areas undetected by Cellpose, as shown in Fig. 2(d)-Day3,5. Traditionally, object and area detection are based on separate models, and adding an area class could achieve more appropriate coloring.

As shown in Fig. 3, class classification analysis highlighted the differences during recovery progression between day 5 CTX and glycerol treatments, yet challenges remain regarding the generalizability of the proposed method. Traditional myofiber classification uses prior knowledge to correlate explicit features (area, circularity, and feret ratio) with desired myofiber types. However, classifying the recovery phase of cells, which have implicit features, requires the expressive power of multidimensional features provided by machine learning methods. Nevertheless, this implicit approach lacks physical explainability, and the classifier results are not necessarily equivalent to human judgments; maintaining accuracy or bridging unforeseen domain gaps in real-world conditions can be challenging. For example, the image in Fig. 3(a)-Day0 should not contain cells in the early phase of regeneration, but due to inadequate freezing during the animal procedure, voids resembling bubbles formed in the myofibers, causing the software to misidentify intact myofibers in stable as early phase (see the Supplementary Fig. S5). Achieving a robust method that can be adapted to a wide range of domains would ideally require broadening the dataset’s domain coverage, but constructing a dataset that accommodates all conditions, such as animal procedures and optical conditions, would require a big effort. While the current implementation of this software cannot guarantee such versatility, it could be utilized in tissue analysis within the specified input image domains.

## Conclusion

We developed MyoRegenTrack to classify and segment cells during muscle tissue recovery. Segmentation was achieved by fine-tuning a pre-trained model, and its robustness was verified by comparing it with models from previous studies. For training the classification model, we used the LLP method based on class proportions associated with date labels, ensuring accuracy comparable to that of experts. We applied MyoRegenTrack to analyze different recovery processes in injections of CTX and glycerol and obtained results consistent with previous studies, confirming its effectiveness. However, the model cannot handle domains not present in the training data, such as poor freezing during specimen preparation when preparing images. In the future, it is necessary to increase the dataset size or explore new data augmentation methods to enhance generalization performance.

## Methods

### Animal procedure

**Fig. 4:**
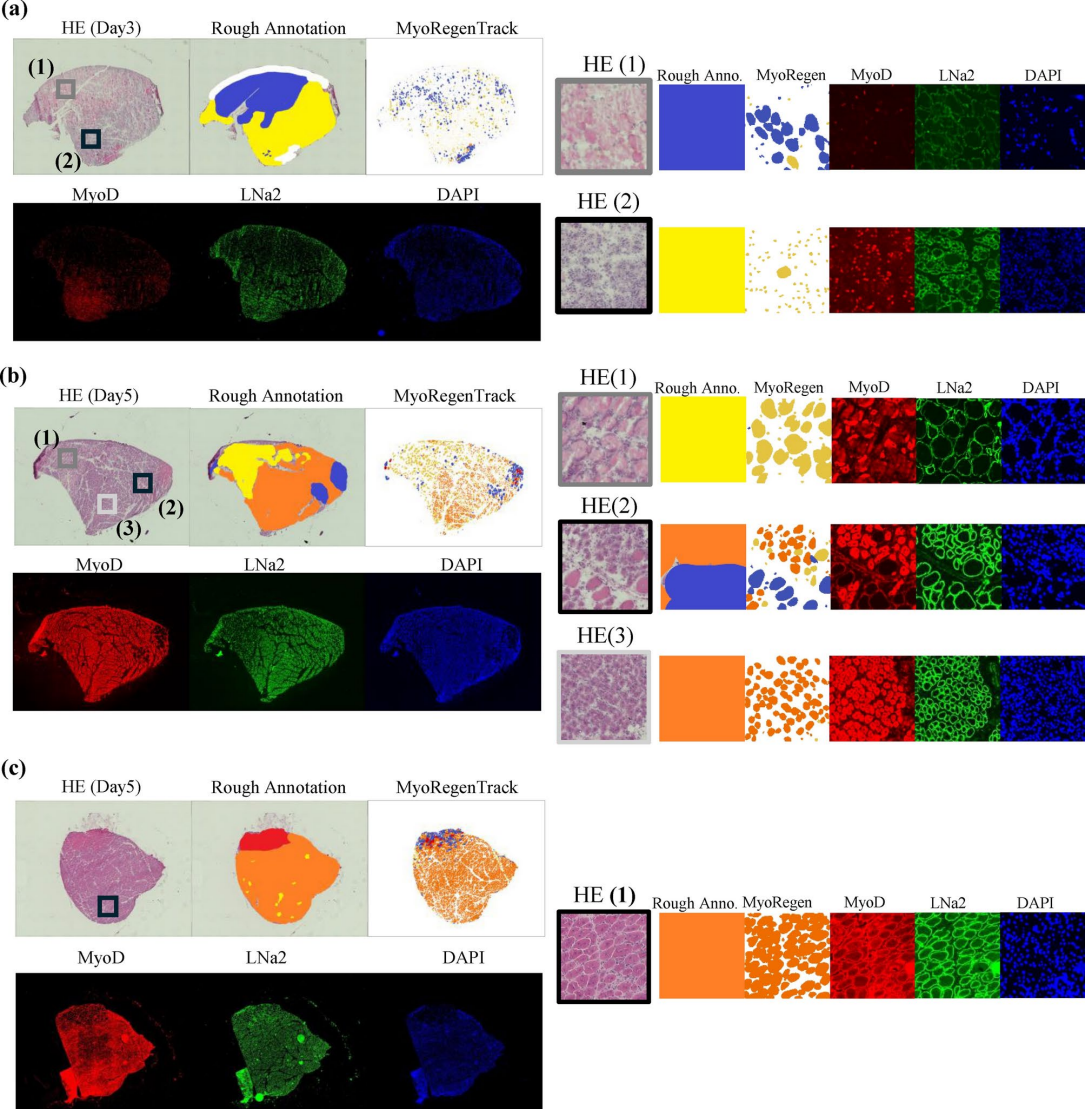
The images for validity of the expert manual annotations. In the rough annotation and MyoRegenTrack, each color corresponds to blue: early phase, yellow: mid-phase, orange: late phase, red: stable, and white indicates areas where no cells were detected. In immunostaining,  (**a**) the red color of Day3 indicates the expression of MyoD,  (**b**) (**c**) the red color of Day5, 7 indicates embryonic myosin heavy chain (eMyHC),  (**a**) (**b**) (**c**) the green color shows the cell membrane marked by Laminin, and the blue color represents the nuclei stained with DAPI.

C57BL/6J mice were purchased from Charles River Laboratories (Yokohama, Kanagawa, Japan). Mice were maintained in a controlled environment (temperature, $24 \pm 2^\circ$C; humidity, $50\% \pm 10\%)$ under a 12/12-h light/dark cycle. The mice were provided sterilized standard chow (DC-8; Nihon Clea, Tokyo, Japan) and water ad libitum. To induce muscle regeneration or degeneration, $100 \mu$L of CTX (Cardiotoxin; $10 {\mu }$M in saline; Latoxan, Valence, France) or 50% v/v glycerol were injected into the tibialis anterior muscles with a 29-gauge needle under anesthesia using a medetomidine, midazolam, and butorphanol cocktail^42–44^. After euthanasia using cervical dislocation by skilled researchers following the Animal Experimentation Committee at Osaka University, tibialis anterior (TA) muscles were dissected, mounted on cork using kneaded Tragacanth Gum (Wako Pure Chemicals Industries, Osaka, Japan), and subsequently flash-frozen in liquid nitrogen-cooled isopentane (Wako Pure Chemicals Industries) for 1 minute. Following a 1-hour incubation on dry ice to evaporate the isopentane, the muscles were stored in sealed containers at $-80^\circ$C. Transverse cryosections were cut $10-{\mu }$m thick and stained with Hematoxylin and eosin solution. In immunostaining of CTX-injected samples for validity of the expert manual annotations, transverse cryosections ($6 {\mu }$m thick) of TA muscles were fixed with 4% paraformaldehyde(PFA) for MyoD (Fig. 4(a)) or cooled-acetone for embryonic myosin heavy chain (eMyHC) staining (Fig. 4(b), 4(c)) for 10 min. After blocking with 5% skimmed milk, adjacent serial sections were stained with primary antibodies at $4^\circ$C overnight. For eMyHC staining, an M.O.M. Kit (Vector Laboratories, Burlingame, CA, USA) was used to block endogenous mouse IgG. The primary antibodies utilized in this study are rat anti-mouse laminin alph2 (Enzo, Clone 4H8-2, Cat# ALX-804-190-C100), rabbit anti-mouse MyoD (Abcam, Cat# ab133627), mouse-eMyHC (DSHB, Clone F1.652), and rabbit anti-collagen type I (Bio-Rad, #2150-1410) antibodies. After washing, the sections were incubated with secondary antibodies conjugated with Alexa Fluor 488, 546, or 647 (Molecular Probes, Eugene, OR, USA). The washed samples were enclosed with VECTASHIELD Mounting Medium with DAPI (Vector Laboratories, #H-1200). To ensure thickness accuracy, we discarded the first or first two slices when changing the thickness of the tissue sections. The stained tissue images were captured using a Plan Apochromat (Keyence, Co) and a 20x objective lens (Nikon, Co.) with a BZ-X Analyzer. The captured image sizes had a width of 2877 to 4606 and a height of 2720 to 4355, totaling 12.5 megapixels. These images were saved in tag image file format (TIFF) along with the information on the elapsed days after CTX injection. Details of our captured images are shown in Table 2. In training data and Day 0 of unlabeled test data, both legs of each mouse were amputated and the tibialis anterior was imaged; otherwise, one image corresponds to a single mouse sample. The images with errors in freezing processing or staining were excluded from the dataset, for example, Day 0 of training data and Day 0 of unlabeled test data. Therefore the training data consists of 14 mice, the annotated test data of 5 mice, and the unlabeled test data on injection of CTX or glycerol consists of 26 mice. All procedures used for experimental animals were approved by the Experimental Animal Care and Use Committee of Osaka University (approval number: R02-3), and all of the methods were carried out under relevant guidelines and regulations and ARRIVE guidelines.

**Table 2:** Number of HE stained images used in experiments in Result section. Image size is width: 2877-4606 [pix], height: 2720-4355 [pix]. The training data was used to finetune Cellpose and train the classifier model. In the annotated test data, class annotations were manually performed by specialists, categorizing the recovery phase of each tissue region into stable (red), early phase (blue), mid-phase (yellow), and late phase (orange) to evaluate the trained model (Fig. 2).

Purpose	Injection	Day0	Day3	Day5	Day7	Day14
Training data	CTX	5	6	6	6	3
Annotated test data	CTX	1	1	1	1	1
Unlabeled test data	CTX	3	3	3	3	3
Unlabeled test data	Glycerol	0	3	3	3	3

### Image preprocessing and dataset creation

We divided all the whole slide images (WSI) into training and test data as shown in Table 2. We created the annotated test dataset by selecting one image each from day 0, 3, 5, 7, and 14 (day 0 refers to the preinjection). We performed expert class manual annotation using Labelbox by categorizing the fiber conditions into four phases: stable (red), early phase (blue), mid-phase (yellow), and late phase (orange)^45^. Red represents intact or fully recovered myofibers. Blue indicates an area of early regeneration characterized by non/low MyoD expression, and basal lamina, but no nuclei as shown Fig. 4(a). Yellow marks the middle phase of regeneration characterized by notable MyoD expression as shown Fig. 4(b). Orange indicates the late phase including small (eMyHC-high) and large myotube (eMyHC-low), both of which have central myonuclei as shown Fig. 4(c). The validity of this manual class annotation provided by visual inspection of specialists and the accuracy of MyoRegenTrack is confirmed by comparing protein markers^45^ obtained from immunostaining of serial sections in Fig.  4. For more detailed information, see Supplementary figures S6, 7, 8.

**Fig. 5:**
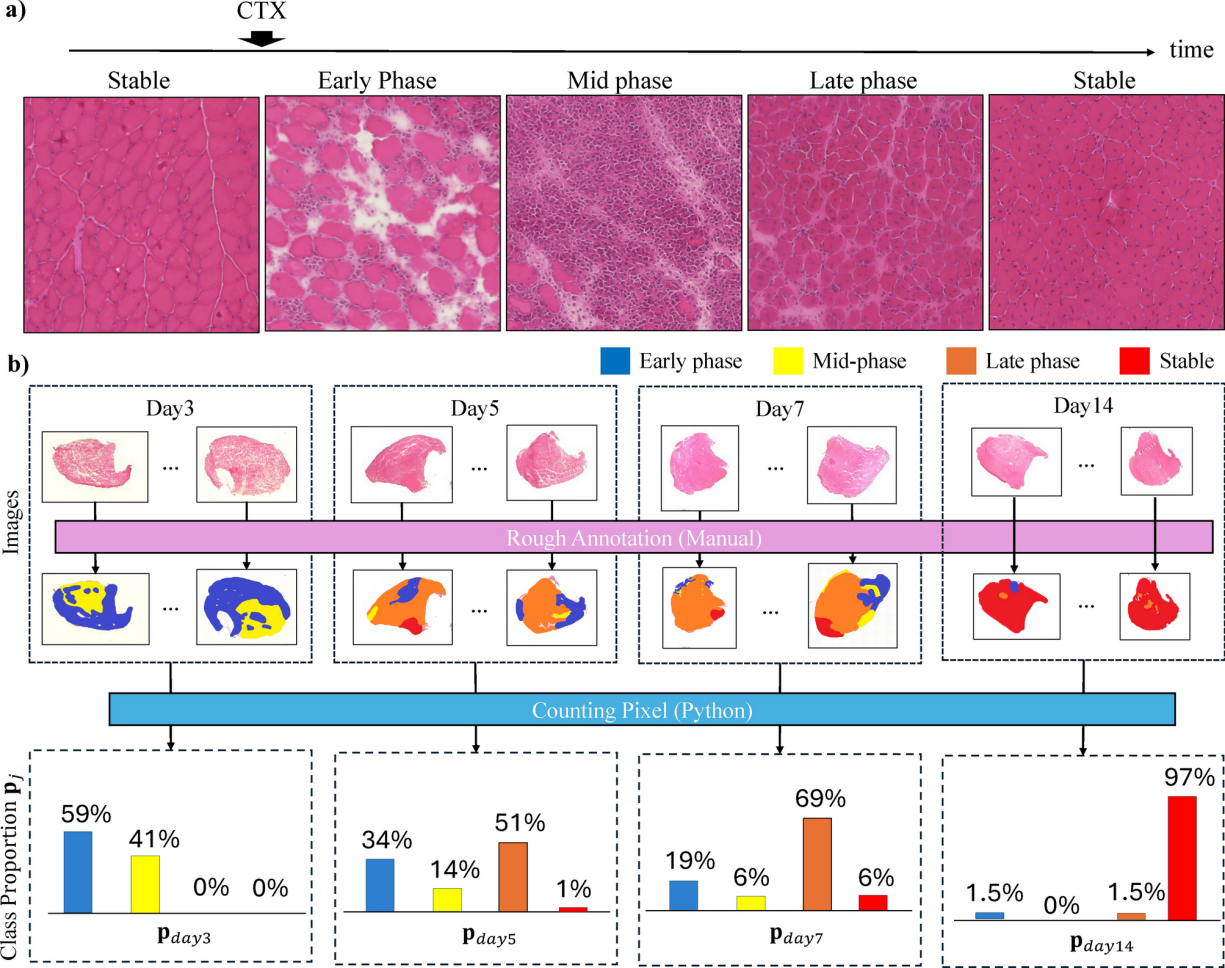
The method for calculating the class proportions corresponding to the daily label. Note that Day 0 is before the CTX injection, and all fibers are considered intact if an image is 100% red. (**a**) The recovery progress of each tissue region after CTX injection. (**b**) Procedure for calculating proportions of each day from the training data based on rough annotation and pixel counting software.

Areas where decisions could not be made were left unannotated and marked in white. Regions outside the muscle tissue were also masked in white. We split the WSIs into grids of $256 \times 256$ [pixel] images during the training and inference processes based on GPU memory limitations. All $256 \times 256$ [pixel] images for training were augmented by rotating them by 90, 180, and 270 degrees using OpenCV 4.8.1 (Python 3.7.13) and flipping them using the Flip function, increasing the data eightfold. We also conduct data augmentation, including RandomBrightness (p=0.5), RandomContrast (p=0.5), and RandomGamma (p=0.5) operations, to simulate random optical conditions using albumentations v1.3.1, doubling the number of images. The full data augmentation process increased the number of images by 16. To train the classifier, we randomly cropped 100 images of $64 \times 64$ [pixel] per grid image ($256 \times 256$ [pixel]) to ensure proper alignment with the roughly annotated proportions shown in Fig. 5.

### Finetuning of cellpose for cell segmentation

As prior cell segmentation methods do not show satisfactory generalizability to skeletal muscles in the recovery process, we finetuned a Cellpose model^46^ to segment all cells that appeared during recovery. We split the WSIs in the training dataset into grids of $256 \times 256$ [pixel] images and manually annotated cell edges in every grid image using the GUI provided by Cellpose^40^, and remove the images that contain no cells. We used the annotated training data to finetune the *cyto2* model of Cellpose version 2.0.3. Channels are set to [0, 0] during finetuning, indicating that cells were identified in grayscale and no nucleus channel was utilized. Other parameters were set to their default settings.

### Computing the proportion of recovery phase on different days

During recovery of skeletal muscle tissues, damaged-myofiber-derived factors (DMDFs) produced from damaged myofiber activate satellite cells, which undergo proliferation and differentiation to regenerate the myofibers^3^. This process can be divided into four phases in terms of recovery phase: stable (denoted as red), early phase (denoted as blue), mid-phase (denoted as yellow), and late phase (denoted as orange), as shown in Fig. 5(a). We define the recovery phase as classes $C = 1,...,k,...K$(i.e. “stable”, “early”, “mid”, and “late”) and manually perform rough class annotation for the training data in Table. 2 by using the Windows application Paint (version 11.2404.45.0). As an index of roughness, the annotations were made with a circular pen of at least 100 pixels in the Paint application preinstalled in Windows. These annotations were intended to determine class proportions and, therefore, cannot serve as an accurate classification of each fiber to evaluate our classifier. We counted the number of pixels in each class from the rough annotations and summed the total pixels per daily label to determine the class proportion for each day in the training dataset. We obtain the proportion $\textbf{p}_j = [p_1,...p_k,...p_K] \in [0,1]^K, \Vert \textbf{p}_j\Vert _1=1$ for classes *C* for each day $j \in \{``day0\text {''}, ``day3\text {''}, ``day5\text {''}, ``day7\text {''}, ``day14\text {''}\}$ by counting the number of pixels in each class from the obtained rough annotations and aggregating the total number of pixels per daily label, as shown in Fig. 5(b).

### Inference pipeline

**Fig. 6:**
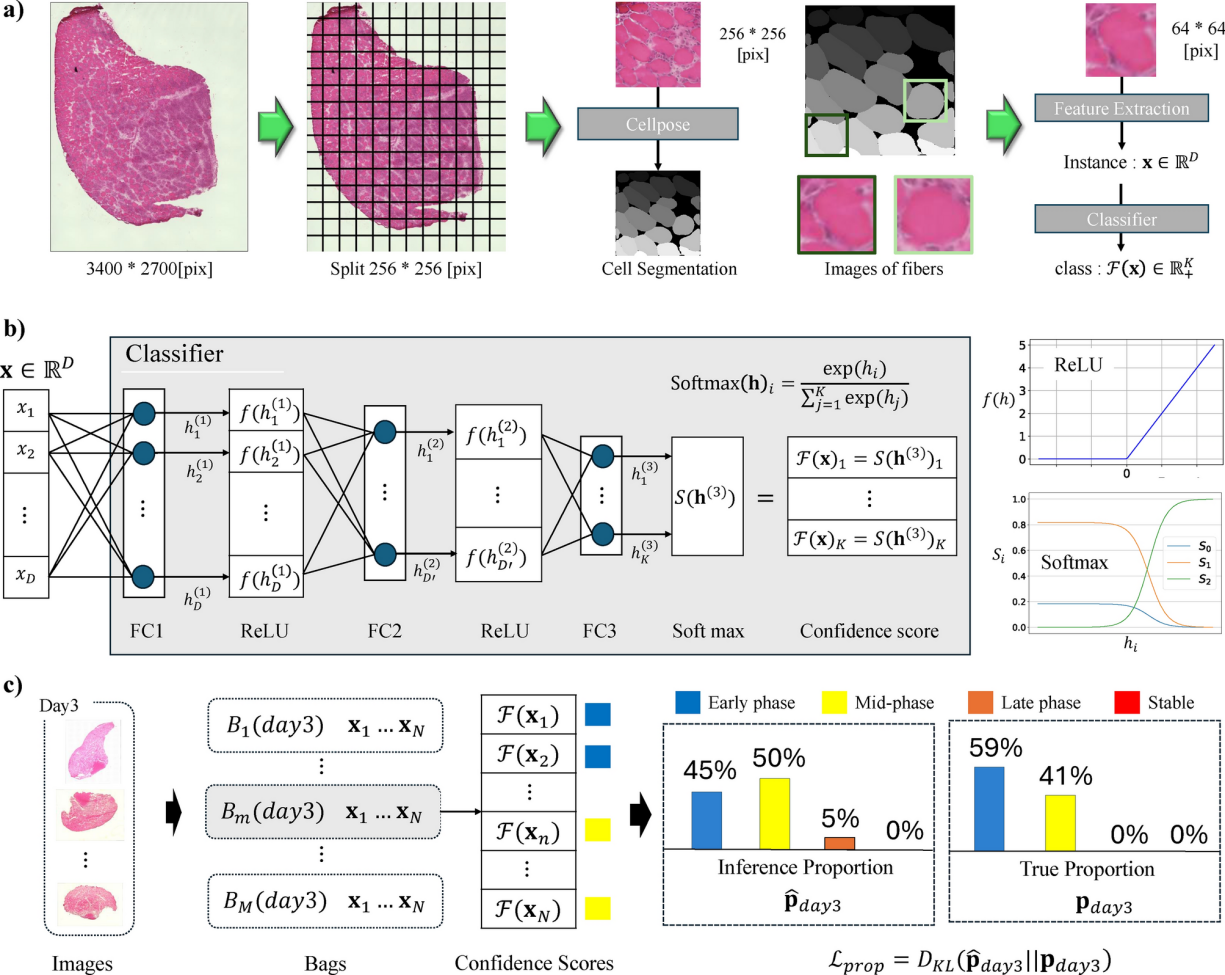
We describe the inference pipeline for the recovery phase classification of all cells from a WSI input in (**a**) and illustrate the architecture of the recovery phase classifier in (**b**). The learning process in the class proportion strategy for training our classifier is illustrated in (**c**).  (**a**) Due to the high resolution of WSIs, it is difficult to perform segmentation directly with Cellpose, so we split the images into $256 \times 256$ [pixel] grids. We calculated the centroid coordinates of each cell using the segmentation result from the finetuned Cellpose and cropped a $64 \times 64$ [pixel] image for each cell. These cell images were passed to a feature extractor to obtain the image features $\textbf{x}\in \mathbb {R}^D$($D \in \mathbb {N}$). The obtained image features were then fed into a classifier implemented based on a three-layer perceptron, which derived the confidence scores for each recovery phase of cells. The class with the highest confidence scores is the predicted recovery phase of the cell.  (**b**) The architecture design of the classifier. An image feature vector of dimension *D* was passed to the first fully connected layer of the classifier (FC1) and processed with a ReLU activation function. It was then passed to the next fully connected layer, FC2, and was compressed to dimension $D'$. After applying another ReLU function, the features were input into the FC3 layer, which outputs a vector of dimension *K*. We applied a Softmax function to the *K*-dimensional vector output to obtain the confidence score for each recovery phase. The class with the highest confidence score is the predicted recovery phase.  (**c**) Our training pipeline using the LLP strategy. We randomly cropped 100 images of $64 \times 64$ [pixel] per grid image. We divide all cropped images into several bags, each containing *N* images. Note that each bag contained only images from the same day. Subsequently, we obtained the confidence scores of all images. We computed the sample proportion of each bag by calculating the sum of the confidence scores and minimized the Kullback-Leibler divergence of the ground truth proportion shown in Fig. 5 from the sample distribution.

We describe the inference pipeline illustrated in Fig. 6(a). An overview diagram for the user is provided in Supplementary Fig. S9. WSIs are split into grids of $256 \times 256$ [pixel] before cell segmentation with Cellpose. The grid image size is adjustable and not critical to the classification accuracy, as it is only used to facilitate cell segmentation using Cellpose. After segmentation, we obtain the contours and centroids of each cell object. For each cell object, we crop a $64 \times 64$ [pixel] image centered on the cell. In this paper, we refer these $64 \times 64$ [pixel] images as “cell images.” We extract the image features of cell images with a generic feature extractor, DINO^24^, to obtain feature instances $\textbf{x}\in \mathbb {R}^D$($D \in \mathbb {N}$: the dimension of the image feature map). We use the DINO ViT-B/8 model, a vision transformer backbone model with a patch size of 8. Additionally, we use the student checkpoint and enable average pooling patch tokens. The other parameters are set to their default settings. The obtained image features are passed to a trained classifier to predict the corresponding recovery phase of cells.

We describe the architecture of our classifier used to predict classes $C = {1, ..., k, ... K}$ (e.g., red, blue, yellow, and orange) from image features $\textbf{x}\in \mathbb {R}^D$. As shown in Fig. 6(b), the classifier learns the mapping $\mathcal {F}: \mathbb {R}^D \rightarrow \mathbb {R}^K_+ (\sum _{k=1}^{K} \mathcal {F}(\textbf{x})_k = 1)$. It consists of a 3-layer perceptron, where we add a rectified linear unit (ReLU^27^) activation function between two fully connected layers. The *K*-dimensional output of the classifier is normalized using a SoftMax function to ensure that the sum of the outputs equals 1, thus representing the confidence of each class.

Pseudo-Label^30,47^ is a weakly supervised learning method in which the daily label *j* is used to establish the class probability distribution $\textbf{p}_j \in [0,1]^K, \Vert \textbf{p}_j\Vert _1=1$. When an instance $\textbf{x}\in \mathbb {R}^D$ with daily label *j* is given, it generates a random class *C* is generated a one-hot vector $\textbf{y}\in \{0,1\}^K$ according to the probability $\textbf{p}_j \in [0,1]^K$. We update the model by calculating the loss $\mathcal {L}_{pseu}$ using cross-entropy for each batch size, as in the equation below.

1 $$\begin{aligned} \mathcal {L}_{pseu} = \sum \mathcal {F}(\textbf{x}) \cdot \log \textbf{y}\qquad \qquad (\because \textbf{y}\in \{0,1\}^K, \ P(y_k=1) = p_k) \end{aligned}$$

We train our classifier model using learning from label proportion (LLP)^31^. LLP is a weakly supervised learning method used to predict the class of each instance given the proportions of bags of instances. In our research, we construct bags by sampling images from the same day and use the proportion of recovery phases on the corresponding day ( Fig. 5) as our ground truth proportion. We optimize our classifier model with a proportion loss $\mathcal {L}_{prop}$, which calculates the KL divergence $D_{KL}$ : $\mathbb {R}^K \cdot \mathbb {R}^K \rightarrow \mathbb {R}_+$ of the ground truth proportion $\textbf{p}_j \in [0,1]^K$ is calculated from the predicted proportions $\hat{\textbf{p}}_{j} \in [0,1]^K$ as follows.

2 $$\begin{aligned} \mathcal {L}_{prop} = D_{\text {KL}}( \textbf{p}_j \Vert \hat{\textbf{p}}_{j}) = \sum _{k=1}^{K} p_k \log \left( \frac{p_k}{\hat{p}_k}\right) \end{aligned}$$

As illustrated in Fig. 6(b,c), we compute image features $\textbf{x}\in \mathbb {R}^D$(*D*: the dimension of the image feature map) related to daily labels *j* from each WSI in the procedure of Fig. 6(a). We are grouping many instances $\textbf{x}$ to *M* bags $\mathbb {B}_1,...,\mathbb {B}_m,...,\mathbb {B}_M$, which has *N*(Bag Size) instance group as $\mathbb {B}_m=\{\textbf{x}_1, \textbf{x}_2,...,\textbf{x}_N\}$ with class proportion $\textbf{p}_j \in [0,1]^K (\Vert \textbf{p}_{j}\Vert _1=1)$ corresponding daily labels *j*. We calculate the loss function $\mathcal {L}_{prop}$ for each bag therefore, the predicted proportions $\hat{\textbf{p}}_{j}$ are computed for each bag. From each instance $\textbf{x}\in \mathbb {R}^D$ within the bag $\mathbb {B}_m$, we obtain the confidence $\mathcal {F}(\textbf{x}) \in [0,1]^K$ through the classifier $\mathcal {F}: \mathbb {R}^D \rightarrow \mathbb {R}^K_+$. The predicted distribution $\hat{\textbf{p}}_{j}=[\hat{p}_1,...,\hat{p}_k,..., \hat{p}_K] \in [0,1]^K, \Vert \hat{\textbf{p}}_{j}\Vert _1=1$ of classes $C={1,...,k,...K}$ (e.g., red, blue, yellow, and orange) for the bag $\mathbb {B}$ is calculated as the average of these confidences values $\mathcal {F}(\textbf{x})$ as follows.

3 $$\begin{aligned} \hat{p}_k = \frac{1}{|\mathbb {B}|} \sum _{\textbf{x}\in \mathbb {B}} \mathcal {F}(\textbf{x})_k \qquad \qquad \qquad (\because \sum _{k=1}^{K} \hat{p}_k = 1) \end{aligned}$$

### Recovery score and cell area rate

As shown in equation (4), the recovery score is calculated by weighting the predicted proportion $\hat{\textbf{p}}_{j}$ with the weight $\varvec{\omega }$.

4 $$\begin{aligned} \textrm{RecoveryScore} = \hat{\textbf{p}}_{j} \cdot \varvec{\omega } =\sum _{k=1}^{K} \hat{p}_k \cdot \omega _{k} \quad (\because \Vert \hat{\textbf{p}}_{j}\Vert _1=1, \ \varvec{\omega } \in [0,1]^K) \end{aligned}$$

The tissue’s recovery score over dates is modeled using the sigmoid function $\sigma (x) = \frac{1}{1 + e^{-a(x-d)}}$, where the gain $a > 0$ represents the slope of the sigmoid curve, reflecting the speed of recovery, and the inflection point $d \in \mathbb {N}$ [day] corresponds to the date when switched to recovery. For this dataset, we set $a = 0.65$ and $d = 6$ [day]. Using true proportion $\textbf{p}_j \in [0,1]^K, \Vert \textbf{p}_j\Vert _1=1$ obtained individually for each WSI in Fig. 5, we derived the weights $\varvec{\omega } \in [0,1]^K$ through the least squares method using Numpy v1.20.3 with the function linalg.lstsq($\textbf{p}_j, \sigma (j)$).

### Statistical analysis

In Fig. 1(d,e), the sample size for the Mean IoU is the number of cells on each date, and the F1 score is the number of $256 \times 256$ [pixel] images about 100-124 segmented from the WSI. Since the Kolmogorov-Smirnov test could not confirm normality, non-parametric tests were conducted to derive p-values and effect sizes. The p-values from the Mann-Whitney test, the effect sizes with Cliff’s delta, and their confidence intervals were calculated using the Python library scipy.stats (version 1.7.3). The graphs were plotted using the Python library matplotlib.pyplot (version 3.5.1).

## Supplementary Information

Supplementary Information.

## Data Availability

The datasets generated during the current study are available from the corresponding author on reasonable request.

## Code availability

Source codes can be found in this Github, and updates will be made periodically.
