# Supplementary material for: Quantifying the recovery process of skeletal muscle on hematoxylin and eosin stained images via learning from label proportion
Source: Sci Rep. 2024 Nov 7;14:27044. doi: 10.1038/s41598-024-78433-z (PMC11544229; doi:10.1038/s41598-024-78433-z)
Supplement: Supplementary file 1 — Supplementary Information. [file 41598_2024_78433_MOESM1_ESM.pdf]

Quantifying the Recovery Process of Skeletal Muscle on Hematoxylin and Eosin Stained Images via Learning from Label Proportion
Supplementary Material

Yu Yamaoka[1], Weng Ian Chan[1], Shigeto Seno[1,*], Kanako Iwamori[2], So-ichiro Fukada[2], and Hideo Matsuda[1]

[1]Graduate School of Information Science and Technology, Osaka University, Osaka, 565-0871, Japan
[2]Graduate School of Pharmaceutical Sciences, Osaka University, Osaka, 565-0871, Japan
[*]senoo@ist.osaka-u.ac.jp

Classifier of SVM by 4 features

To supplement the classification performance discussed in the introduction using a Support Vector Machine (SVM)

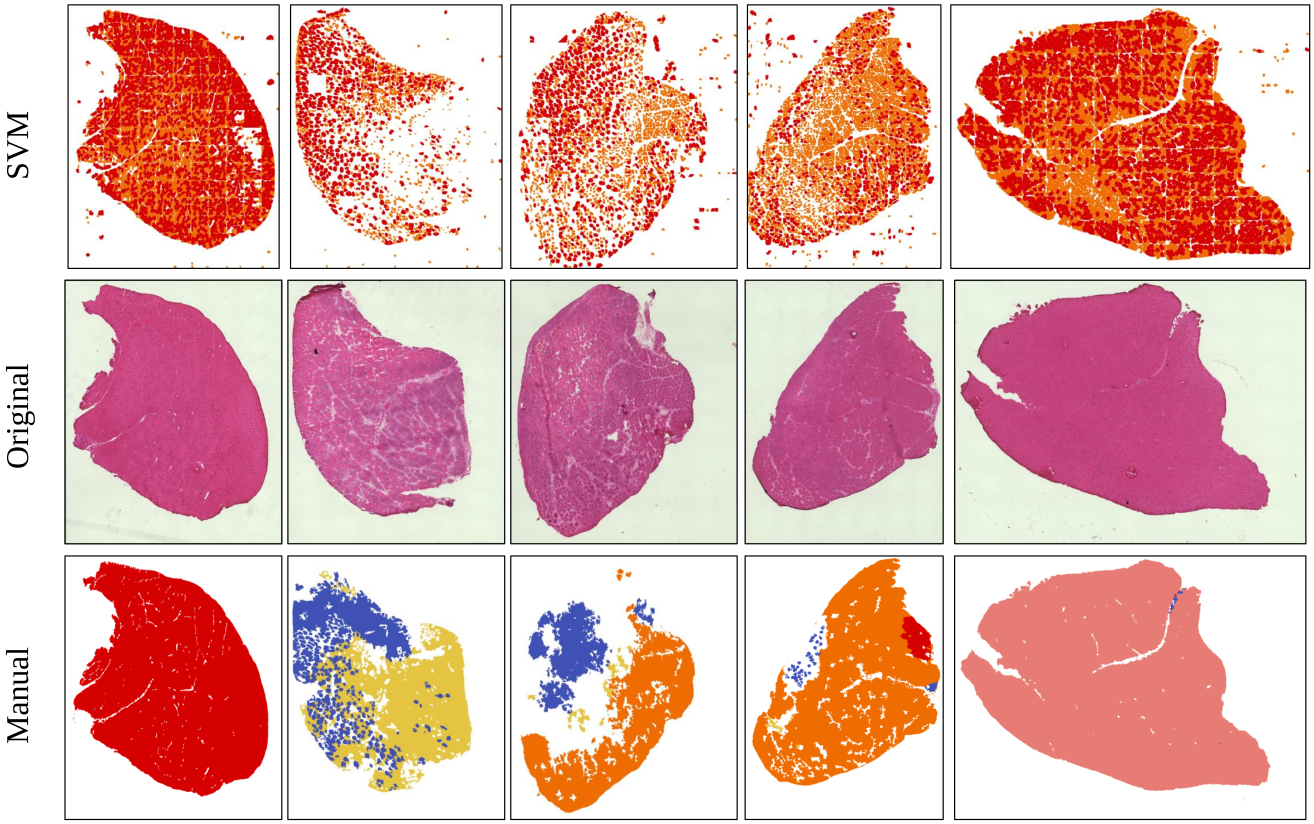

Figure S1. The classification results using a Support Vector Machine (SVM) are presented. The results are based on four features—Area, Circularity, Minimum Feret, and Feret Aspect Ratio—obtained from the segmentation in Myosoft[1], along with the date proportion data to generate pseudo-labels for SVM training and inference. The colors correspond to Red: Myofiber, Yellow: Small Myotube, Blue: Myoblast, and Orange: Large Myotube

Applying software for laminin to HE-stained images

The results of applying OpenCSAM[2], developed for laminin-stained images, to HE-stained images are presented.

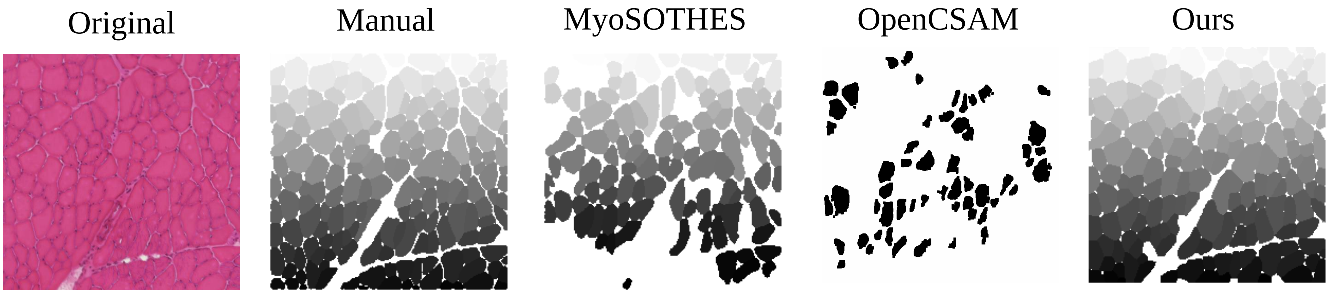

Figure S2. The input consists of 256 × 256 [pixel] images. The manual refers to segmentation results performed manually. The parameters for MyoSOTHES and Ours are the same as described in the main text. For OpenCSAM, the default parameters were used.

Recovery of glycerol injection

For the glycerol samples of Day 7 and 14 muscles as shown Fig. S3, after 4% PFA fixation, adjacent serial sections were stained with either anti-LNa2 and -Perilipin antibodies or anti-LNa2 and -Collagen I antibodies. Nuclei were counterstained with DAPI. The imaging method is the same as described in the main text's "Animal Procedure" section. We performed level adjustment and gamma correction (γ =0.7) on the fluorescent-stained images for visibility. The input to MyoRegenTrack uses the original captured HE-stained images.

As seen from the results of Fig. S3, it can be observed that inflammation occurs in certain areas of the tissue as days progress since glycerol injection. Perilipin indicates adipogenesis, and collagen indicates the occurrence of fibrosis. In other words, tissue injected with glycerol does not exhibit a recovery trend like tissue injected with CTX.

Comparison of glycerol and CTX by using Pseudo-Label

Evaluate the recovery progress of mice injected with CTX and glycerol using a model trained with suspected labels as shown in Fig. S4. Focusing on the results from Day 5, it becomes apparent that an incorrect Recovery Score might be calculated if an error occurs in the predicted class.

Limitation of our softwares

When inputting the Day 0 images, which should contain only Myofibers, of tissues with inadequate freezing before CTX injection into the proposed software, obvious misclassification results were obtained (Fig. S5). This indicates that the proposed software cannot adequately handle such unexpected domains.

Comparison with immunohistochemical labelling

To verify the validity of the expert manual annotations on HE images used as ground truth labels in this study, and to confirm that glycerol addition induces adipogenesis and fibrosis, we examined fluorescence images of various marker proteins.

For immunostaining of CTX-injected samples, transverse cryosections (6 μm thick) of TA muscles were fixed with 4% paraformaldehyde(PFA) for MyoD (Fig. S6) or cooled-acetone for embryonic myosin heavy chain (eMyHC) staining (Fig. S7, S8) for 10 min. After blocking with 5% skimmed milk, the sections were stained with primary antibodies at 4ºC overnight. For eMyHC staining, an M.O.M. Kit (Vector Laboratories, Burlingame, CA, USA) was used to block endogenous mouse IgG. The primary antibodies utilized in this study are rat anti-mouse laminin alph2 (Enzo, Clone 4H8-2, Cat# ALX-804-190-C100), rabbit anti-mouse MyoD (Abcam, Cat# ab133627), mouse-eMyHC (DSHB, Clone F1.652), and rabbit anti-collagen type I (Bio-Rad, #2150-1410) antibodies. After washing, the sections were incubated with secondary antibodies conjugated with Alexa Fluor 488, 546, or 647 (Molecular Probes, Eugene, OR, USA). The washed samples were enclosed with VECTASHIELD Mounting Medium with DAPI (Vector Laboratories, #H-1200). To ensure thickness accuracy, we discarded the first or first two slices when changing the thickness of the tissue sections.

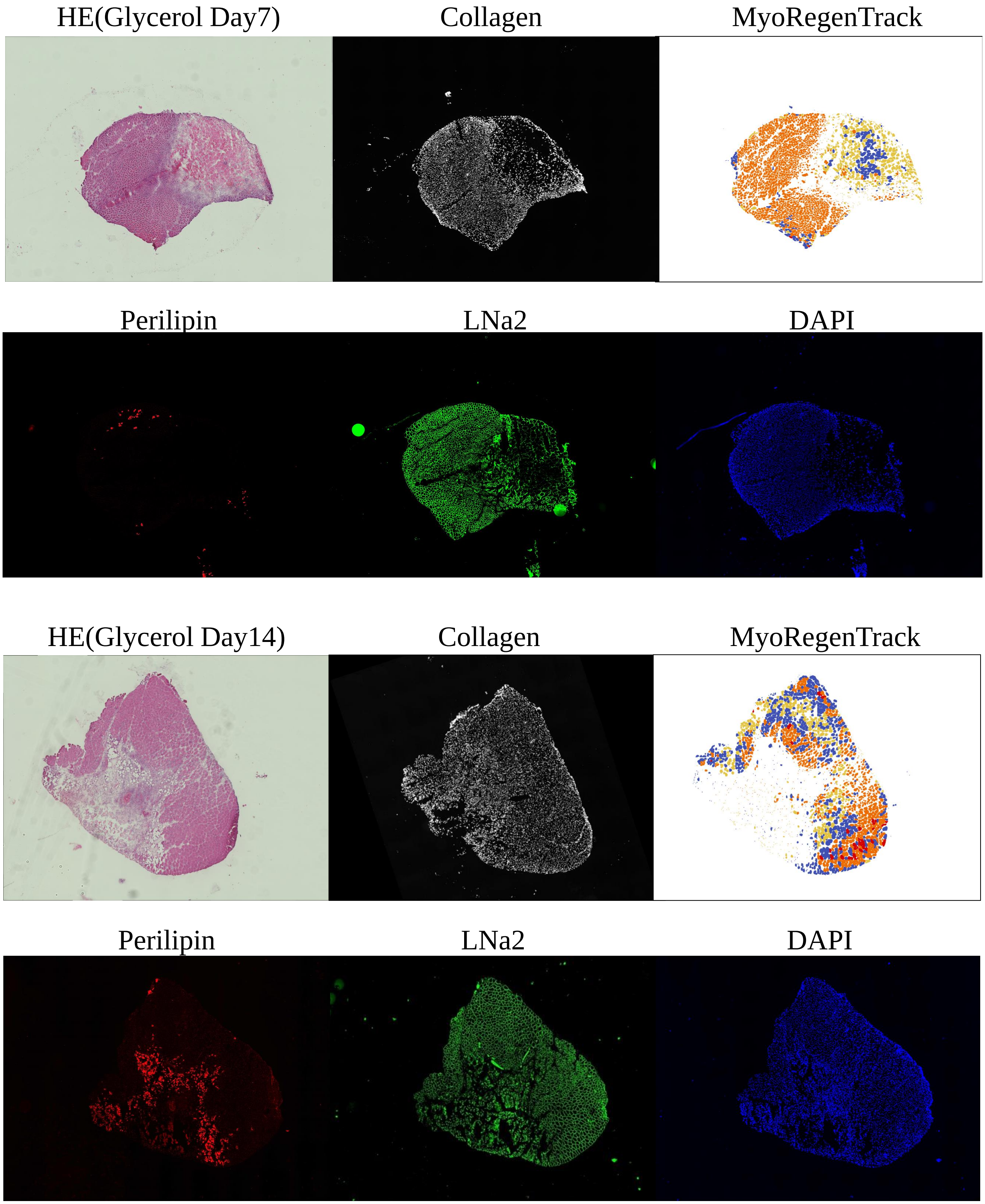

Figure S3. A tissue section after seven or fourteen days has elapsed since Glycerol injection. In the MyoRegenTrack, each color corresponds to blue: early phase, yellow: mid-phase, orange: late phase, and red: stable. In immunostaining, the white color indicates collagen, the red color indicates perilipin, the green color shows the cell membrane marked by Laminin, and the blue represents the nuclei stained with DAPI.

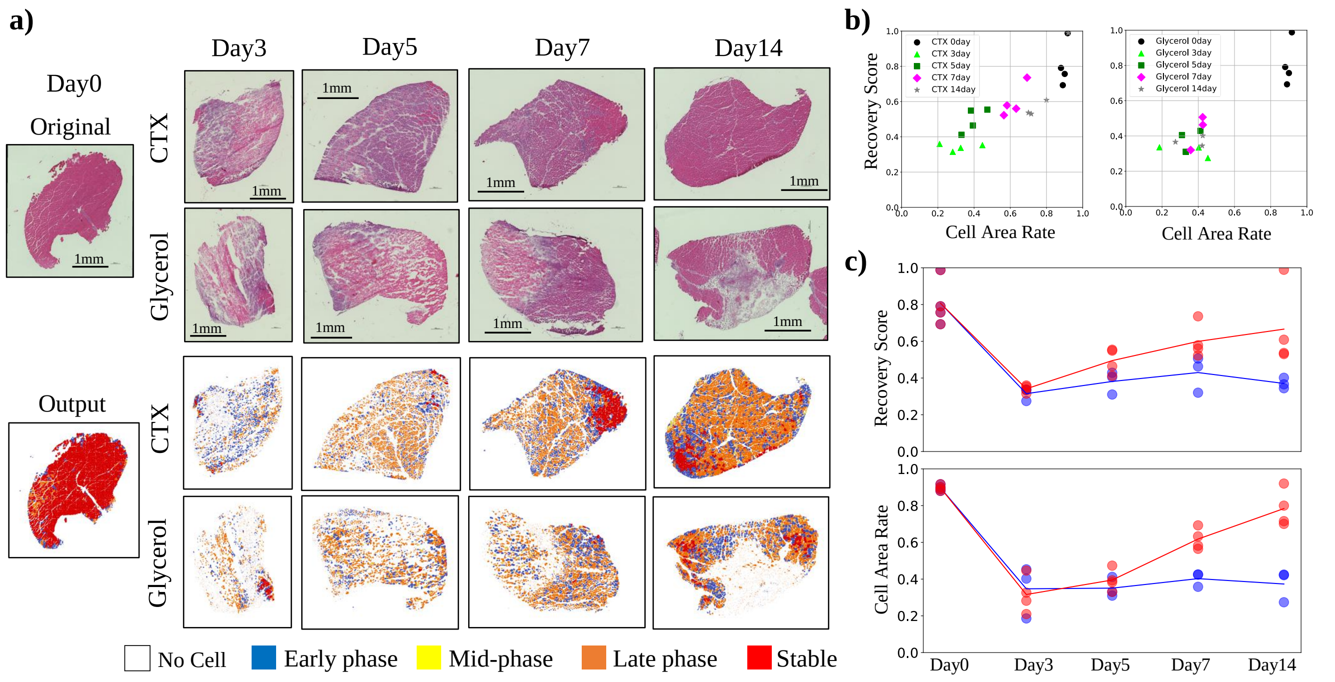

Figure S4. **a)** Day 0 represents the tissue before injection. Days 3 and beyond indicate the number of days elapsed since the injection of CTX or glycerol, which induces necrosis and recovery in the tissue. The output displays the results by pseudo-label model, where each color corresponds to Red: Myofiber, Yellow: Small Myotube, Blue: Myoblast, Orange: Large Myotube, and white indicates areas where no cells were detected.
b, c) We count the pixels of each color within the edge-detected tissue and compute the proportion $\hat{p}$ of each color relative to the Cell Area (Segmentation Area). The Recovery Score was calculated for each image. Each point in this figure corresponds to one WSI. For the Cell Area, the area ratio of cells detected by Cellpose[3] to the muscle tissue region $\frac{SegmentationArea}{StainArea}$ was calculated. Note that since Day 0 is before injection, the data for CTX and glycerol are the same.

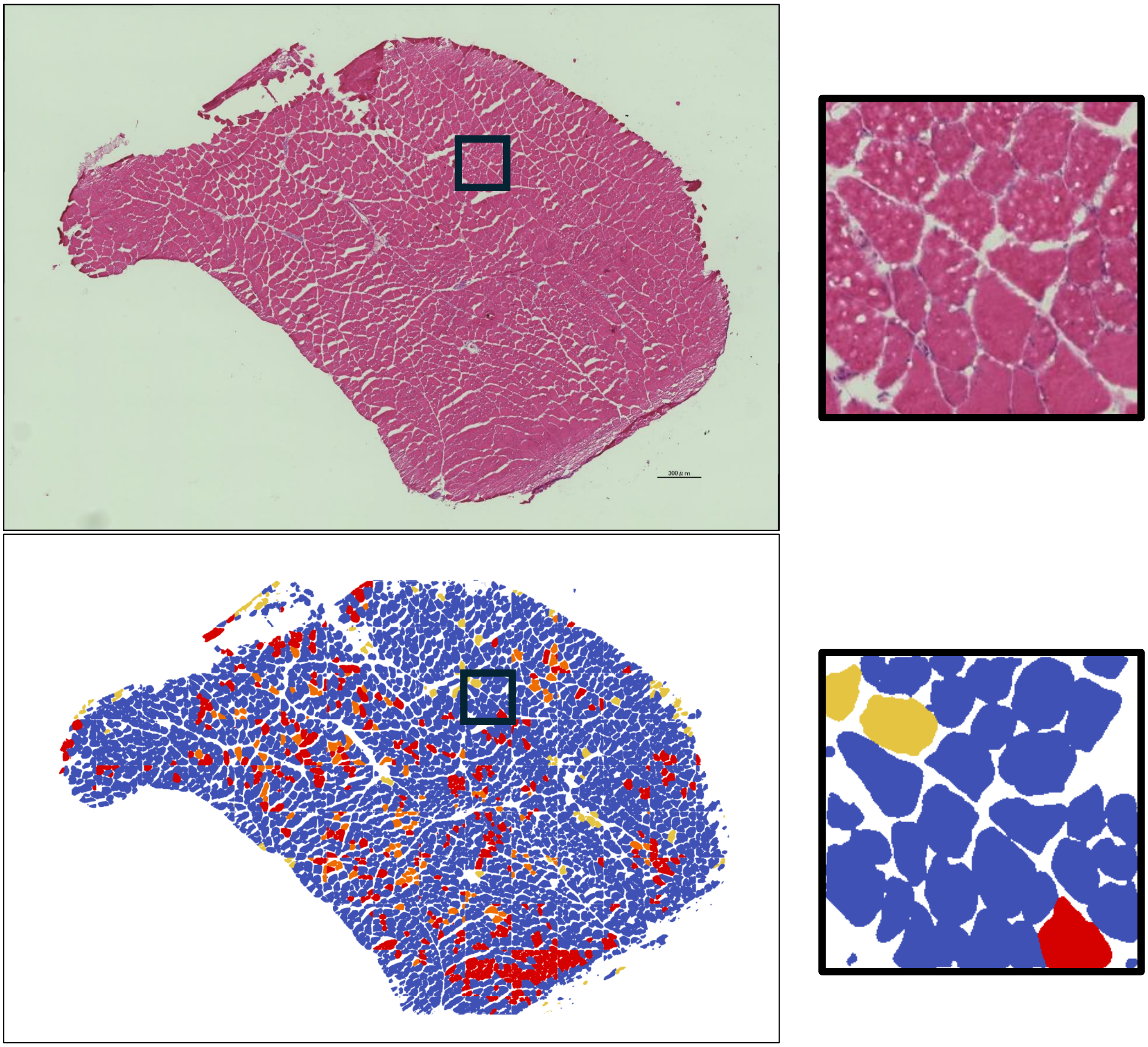

Figure S5. Results of inputting the pre-injection Day 0 images, which had inadequate freezing treatment, into the proposed software. Red corresponds to Myofiber, Yellow to Small Myotube, Blue to Myoblast, and Orange to Large Myotube.

In the rough manual annotation made by visual inspection of specialist and MyoRegenTrack, color results indicate that blue represents an early phase of regeneration, characterized by non/low MyoD expression, and basal lamina, but no nuclei. Yellow represents a mid-phase, which is characterized by notable MyoD expression. Orange represents a late phase including small (eMyHC-high) and large myotube (eMyHC-low), both of which have central myonuclei. As seen from the results of Fig. S6, Fig. S7 and Fig. S8, inference results from MyoRegenTrack and manual annotation of recovery stages based on cell features obtained from HE-stained images were consistent in many regions with the recovery stages estimated from protein markers[4] obtained through immunostaining.

Inference Pipeline

An overview of the proposed software is presented in Fig. S9.

References

1. Encarnacion-Rivera, L., Foltz, S., Hartzell, H. C. & Choo, H. Myosoft: an automated muscle histology analysis tool using machine learning algorithm utilizing fiji/imagej software. *PLOS ONE* **15**, e0229041, DOI: https://doi.org/10.1371/journal.pone.0229041 (2020).

2. Desgeorges, T. *et al.* Open-CSAM, a new tool for semi-automated analysis of myofiber cross-sectional area in regenerating adult skeletal muscle. *Skeletal Muscle* **9**, DOI: https://doi.org/10.1186/s13395-018-0186-6 (2019).

3. Stringer, C., Wang, T., Michaelos, M. & Pachitariu, M. Cellpose: a generalist algorithm for cellular segmentation. *Nat. Methods* **18**, 100–106, DOI: https://doi.org/10.1038/s41592-020-01018-x (2021).

4. Stephens, D. C. *et al.* Protocol for isolating mice skeletal muscle myoblasts and myotubes via differential antibody validation. *STAR Protoc.* **4**, 102591, DOI: https://doi.org/10.1016/j.xpro.2023.102591 (2023).

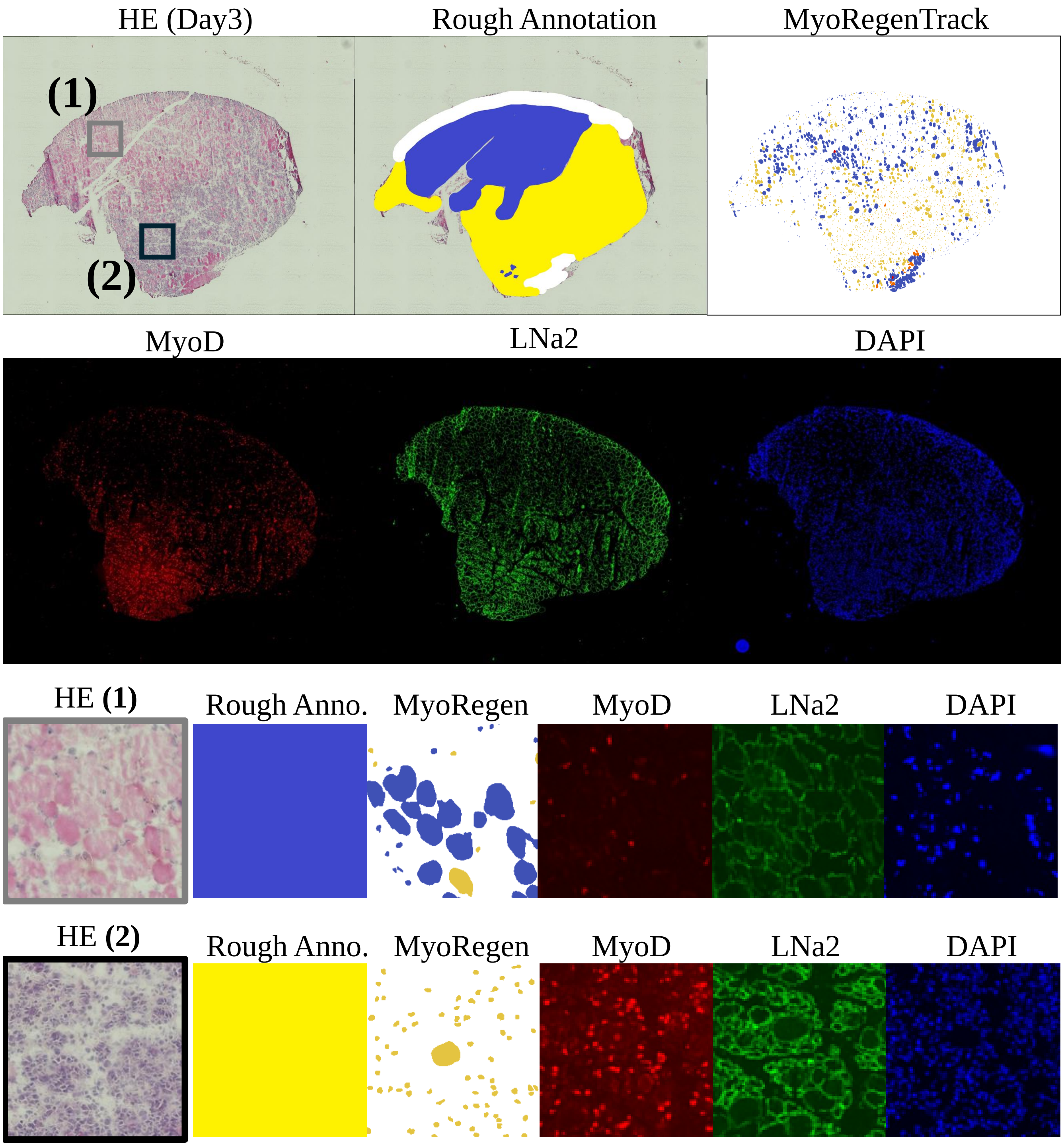

Figure S6. A tissue section after three days has elapsed since CTX injection. In the rough annotation and MyoRegenTrack, each color corresponds to blue: early phase, yellow: mid-phase, orange: late phase, and white indicates areas where no cells were detected. In immunostaining, the red color indicates the expression of MyoD, the green color shows the cell membrane marked by Laminin, and the blue color represents the nuclei stained with DAPI.

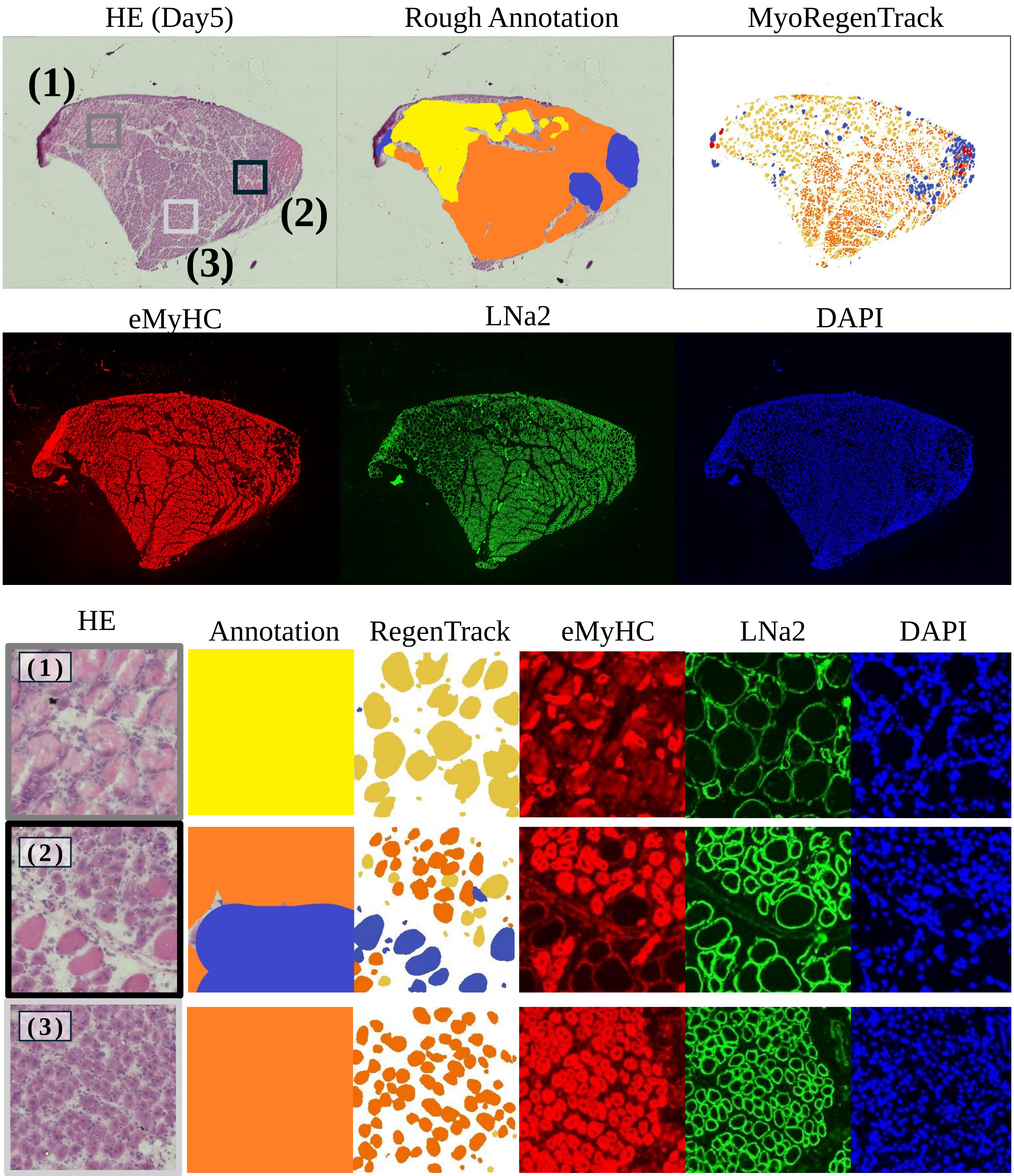

Figure S7. A tissue section after five days has elapsed since CTX injection. In the rough annotation and MyoRegenTrack, each color corresponds to blue: early phase, yellow: mid-phase, orange: late phase, red: stable, and white indicates areas where no cells were detected. In immunostaining, the red color indicates embryonic myosin heavy chain (eMyHC), the green color shows the cell membrane marked by Laminin, and the blue represents the nuclei stained with DAPI.

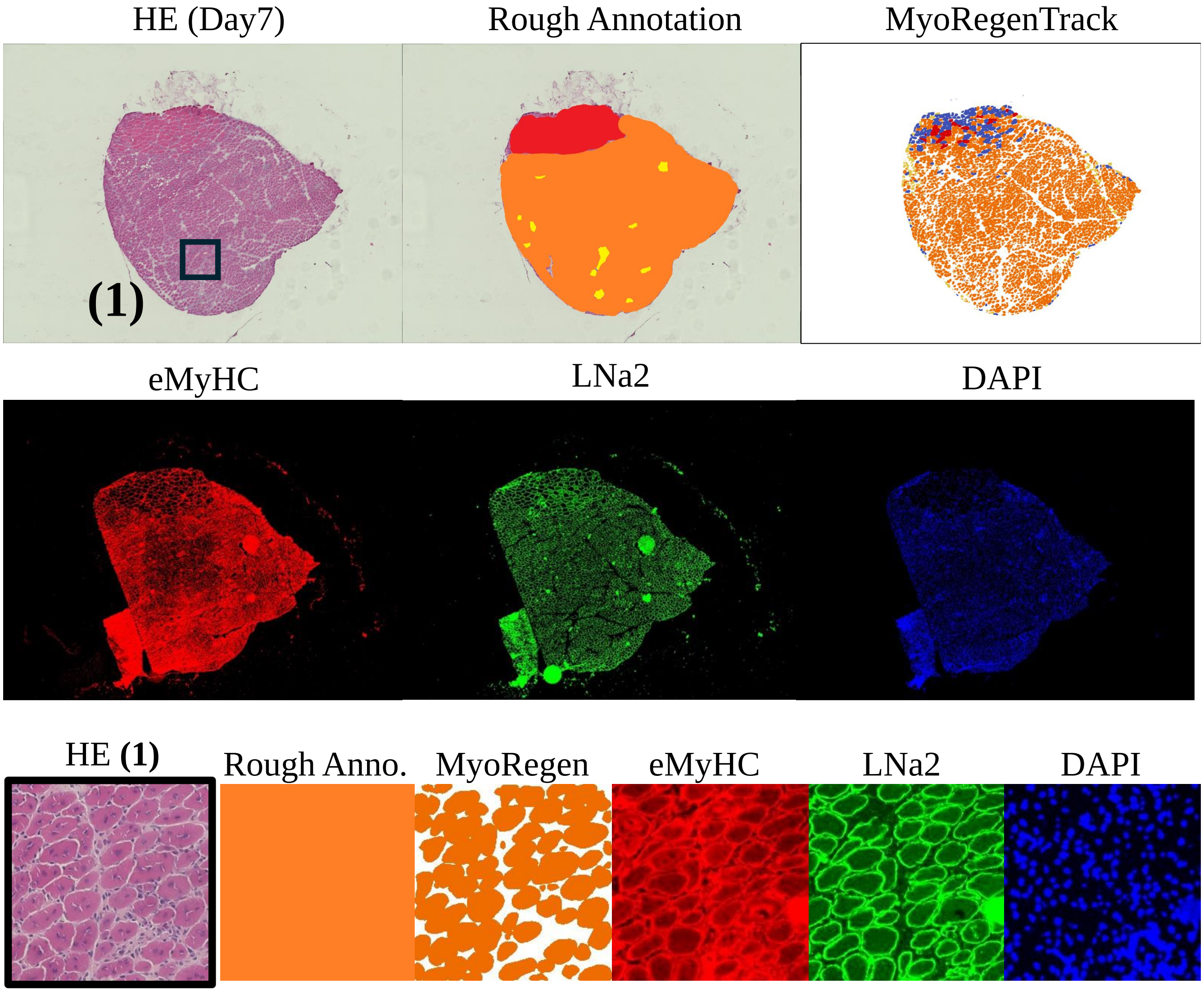

Figure S8. A tissue section after seven days has elapsed since CTX injection. In the rough annotation and MyoRegenTrack, each color corresponds to blue: early phase, yellow: mid-phase, orange: late phase, red: stable, and white areas representing indeterminate regions. In immunostaining, the red color indicates embryonic myosin heavy chain (eMyHC), the green color shows the cell membrane marked by Laminin, and the blue represents the nuclei stained with DAPI.

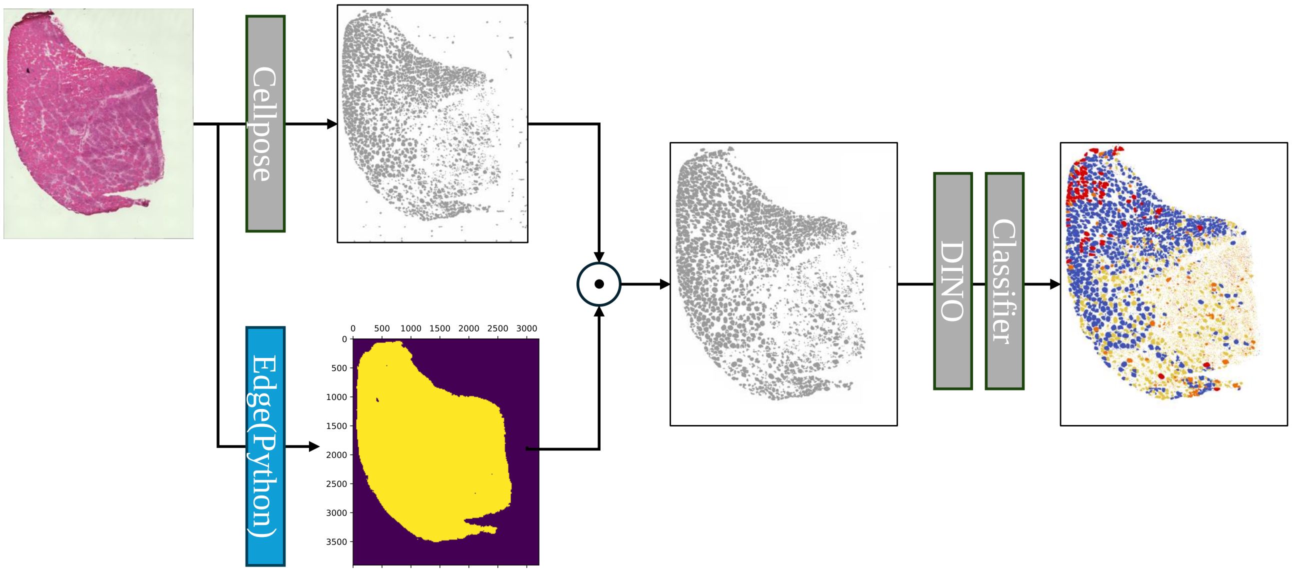

Figure S9. When input into Cellpose, the images are clipped to 256×256 [pixel], and when input into DINO and the Classifier, they are clipped to 64×64 [pixel] according to the procedure in Fig. 5(a) of the main text. Parallel to being input into Cellpose, the input images are processed by Python software for edge detection to extract masks of the stained image regions. The results from Cellpose and the edge detection are combined using the Hadamard product, resulting in segmentation data of only the stained regions. By passing these through DINO and the Classifier, the segmented data for each cell is color-coded according to its class and outputted.
